# The Crystal Structure of the Dachshund Domain of Human SnoN Reveals Flexibility in the Putative Protein Interaction Surface

**DOI:** 10.1371/journal.pone.0012907

**Published:** 2010-09-23

**Authors:** Tomas Nyman, Lionel Trésaugues, Martin Welin, Lari Lehtiö, Susanne Flodin, Camilla Persson, Ida Johansson, Martin Hammarström, Pär Nordlund

**Affiliations:** 1 Structural Genomics Consortium, Karolinska Institutet, Stockholm, Sweden; 2 Department of Medical Biochemistry and Biophysics, Karolinska Institutet, Stockholm, Sweden; 3 Pharmaceutical Sciences, Department of Biosciences, Åbo Akademi University, Turku, Finland; University of Washington, United States of America

## Abstract

The human SnoN is an oncoprotein that interacts with several transcription-regulatory proteins such as the histone-deacetylase, N-CoR containing co-repressor complex and Smad proteins. This study presents the crystal structure of the Dachshund homology domain of human SnoN. The structure reveals a groove composed of conserved residues with characteristic properties of a protein-interaction surface. A comparison of the 12 monomers in the asymmetric unit reveals the presence of two major conformations: an open conformation with a well accessible groove and a tight conformation with a less accessible groove. The variability in the backbone between the open and the tight conformations matches the differences seen in previously determined structures of individual Dachshund homology domains, suggesting a general plasticity within this fold family. The flexibility observed in the putative protein binding groove may enable SnoN to recognize multiple interaction partners.

**Enhanced version:**

**This article can also be viewed as an enhanced version in which the text of the article is integrated with interactive 3D representations and animated transitions. Please note that a web plugin is required to access this enhanced functionality. Instructions for the installation and use of the web plugin are available in Text S1.**

## Introduction

The Ski and SnoN oncoproteins regulate gene expression through their interaction with a number of transcription factors such as Smads [1], [2], retinoic acid receptor α (RARα) [3], [4], and retinoblastoma protein (pRb) [5]. They also interact with transcriptional co-regulators such as the nuclear hormone receptor co-repressor (N-CoR) and silencing mediator of retinoid and thyroid hormone receptors (SMRT), both components of a macromolecular repressor complex containing mSin3 and histone deacetylase (HDAC) [6], [7].

**Figure 1:**
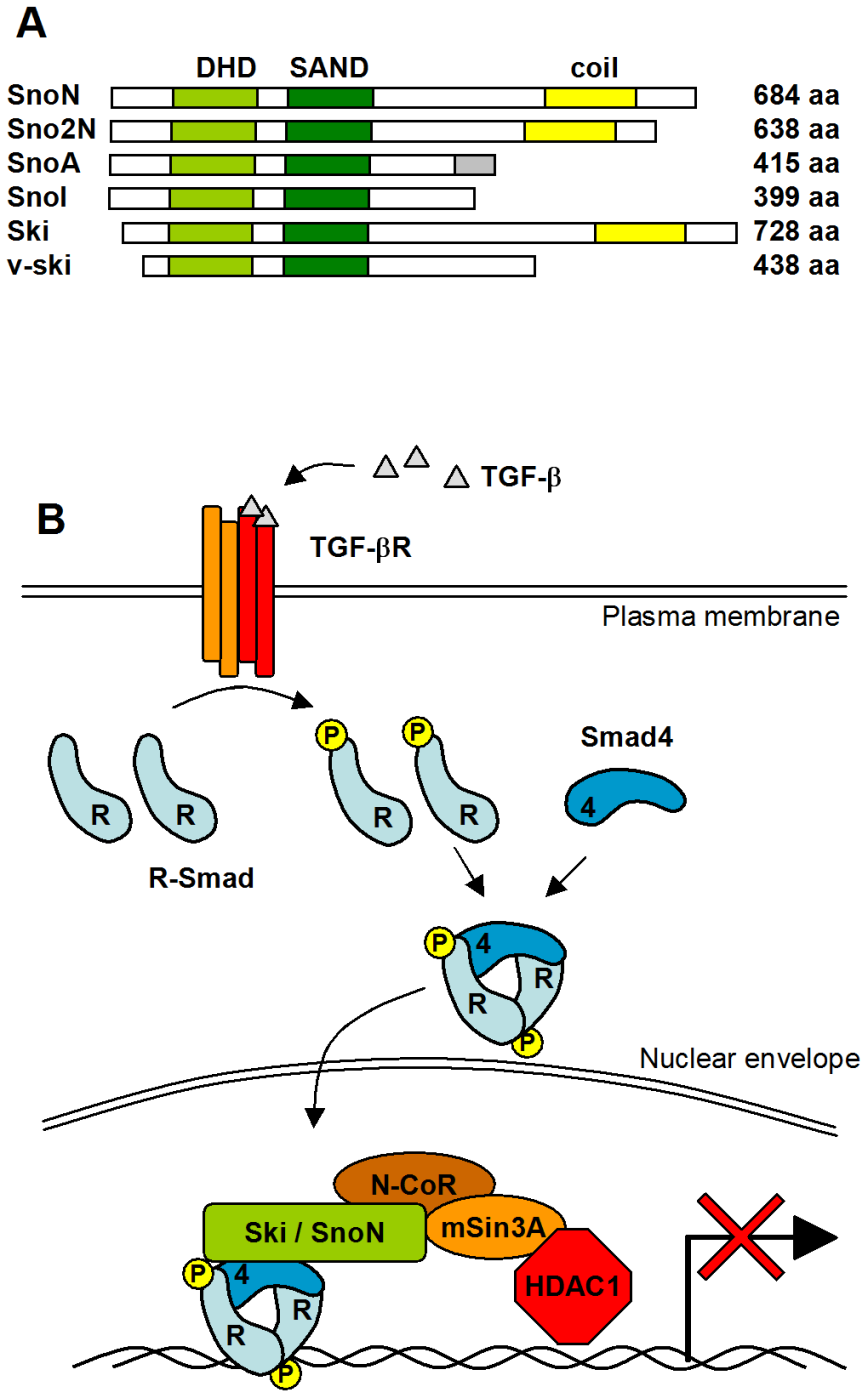
Domain structure and functional overview of the Ski/Sno proteins. **Panel A**. Schematic representation of the Ski/Sno family of proteins with the conserved domains colored. The family includes the full-length SnoN and splice variants from the *sno* gene: SnoN2 with 46 aa deletion in the C-terminal region; SnoA, which carries a shorter and unique C-terminal sequence (grey) compared to SnoN; and SnoI, covering only aa 1–399. Human Ski and the v-ski protein of the Sloan Kettering virus are added for comparison. **Panel B**. Simplified overview of Ski and SnoN involvement in Smad-regulated TGF-β signaling. Ski and Sno proteins can act as bridges between the Smad complex and the N-CoR/mSin3A/HDAC1 repressor complex.

The Ski/Sno protein family is defined by homology with v-Ski, the transforming protein of Sloan Kettering virus [8]–[11]. The *sno* gene (a.k.a *skil* for Ski-like) encodes SnoN protein of 684 residues as well as three splice variants with varying C-terminal parts. The *ski* gene has not been reported to give rise to alternative isoforms. Ski and SnoN share three structural domains, the N-terminal Dachshund homology domain (DHD), a Smad4-binding domain, and a C-terminal coiled-coil domain (Fig. 1A). The DHD is a ∼100 aa globular domain with structural homology to the forkhead/winged-helix family of DNA-binding proteins [12], [13]. The Smad4-binding domain of ∼100 aa shares structural homology with DNA-interacting SAND (Sp100, AIRE1, NucP41/75, DEAF1) domains, found in a number of chromatin remodeling proteins [14], [15].Although, both the DHD and the Smad4-binding domains are related to DNA-binding domains, neither Ski nor SnoN bind DNA but act as protein interaction modules [16]. The C-terminal coiled-coil domain has BAR domain-like features and has been reported to mediate both hetero- and homodimerization of Ski and SnoN [17], [18]. The Ski family also contains two more recently described members: Fussel-18 (functional Smad suppressing element on chromosome 18) [19], and Fussel-15 [20].

Both Ski and SnoN are predominantly nuclear proteins and classified as oncoproteins based on their transformation ability when overexpressed in chicken or quail embryo fibroblasts [21], [22]. They also display increased expression levels in various human tumors [10], [23]. The most studied role of Ski and SnoN is their negative regulation of tumor growth factor beta (TGF-β) induced signaling. Members of the TGF-β superfamily of extracellular growth factors include TGF-β, bone morphogenic protein (BMP) and activin. These growth factors regulate multiple cellular processes, such as cell proliferation, differentiation, apoptosis and developmental fate in metazoans [24]. TGF-β signaling is relayed by members of the Smad family of transcription factors (Fig. 1B). Activated TGF-β receptors recruit and phosphorylate R-Smads (Smad2/3) which then associate with the co-Smad, Smad4, to form the active trimeric Smad complex. This Smad complex is translocated to the nucleus, where it binds Smad-binding elements within promoters of TGF-β responsive genes to regulate their transcription, either by activation or repression [25].

Ski and SnoN have been reported to regulate Smad-mediated signaling through multiple mechanisms. At the promoter site, Ski and SnoN recruit the HDAC repressor complex to Smads, acting as a bridge binding N-CoR through the DH domain [7]. Moreover, the binding of Ski to Smad4 has been reported to destabilize the trimeric Smad complex [15], at the promoter site in the nucleus, as well as in the cytoplasm [26]. In addition, binding of Ski to R-Smads 2 and 3 have been suggested to interfere with their recruitment to the TGF-β receptor complex [15].

This study describes the crystal structure of the Dachshund homology domain of human SnoN. Structure and sequence analysis revealed a highly conserved cleft in the expected protein interaction face, with the hallmarks of a protein-protein interaction interface. The 12 molecules in the asymmetric unit reveal significant flexibility of this region, suggesting the existence of an open and a tight conformation. The variable regions of the fold corresponds to the differences seen between previously published structures of DH domains, *i.e.* from Ski and Dach1 [12], [13]. The flexibility might be necessary for the DH domain to accommodate binding motifs from different interaction partners.

## Results and Discussion

### Overall description of the structure

A fragment of human SnoN encompassing residues 137 to 238, covering the Dachshund homology domain (SnoN-DHD), was crystallized and diffraction data was collected to 2.45 Å. The structure was solved by molecular replacement using the structure of the Dachshund domain of human Ski (Ski-DHD) as a search model (PDB entry: 1SBX) [12]. Statistics of the data processing and refinement of the structure are presented in Table S1. The asymmetric unit in the crystal contained twelve protein molecules and the crystal organization can be viewed as a non-crystallographic trimer of tetramers. During purification SnoN-DHD migrated as a monomer in size exclusion chromatography (data not shown). Atomic models were built for residues 144 to 235 for all chains, and additional termini residues within 141–238 were built for most chains.

**Figure 2:**
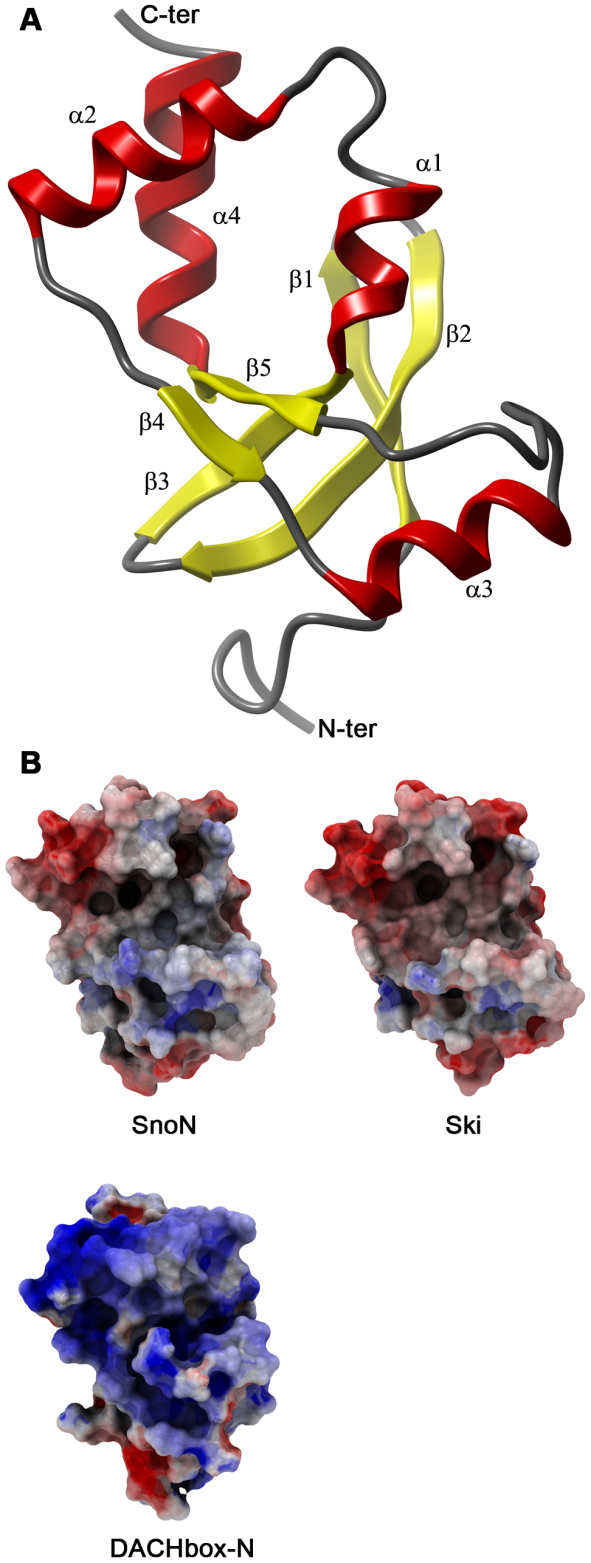
The SnoN-DHD structure and surface charge comparison. **Panel A**: Ribbon representation of SnoN-DHD. **Panel B**: Comparison between electrostatic surfaces of SnoN-DHD (chain A), Ski-DHD and DACHbox-N. Molecular surfaces are colored according to electrostatic potentials with maximum color potential set to ±5 kT/e. SnoN-DHD, Ski-DHD (PDB 1SBX) and DACHbox-N (PDB 1L8R) are in the same orientation as in Figure 2A. Electrostatic potential was calculated with the program ICM-Pro v.3.7-1e [29] using the REBEL (rapid-exact-boundary-element) method.

The SnoN-DHD is a small globular domain which belongs to the α-β roll architecture according to the CATH classification [27]. It is composed of a twisted five-stranded anti-parallel β-sheet sandwiched between three α-helices on one side and one α-helix on the other (Fig. 2A). Helix α3 is separated from helices α1, α2 and α4 by one extended stretch of residues (191 to 198) on its N-terminal side, and a turn, whose specific conformation is conferred by Pro 212, on its C-terminal side. Helix α2 is lined by the 191–198 stretch on its C-terminal side and by a helix-turn-helix on its N-terminal part. The extended conformation of residues 191 to 198 seems to restrict the relative motions of the two helical domains on this side of the molecule and might therefore rigidify the scaffold of SnoN-DHD.

### Comparisons within the DHD family

The DH domain is related to the forkhead/winged-helix type of DNA-binding motifs in which the “wing” is a β-strand hairpin corresponding to strands β4 and β5 of the DHD, and where the “helix” corresponds to the α2 helix of the DHD. In the DH domain, the helix α3 has been inserted in the hairpin loop [13] (Fig. 2A). Due to this apparent kinship, the DH domain was suggested to be DNA binding, and indeed there is evidence indicating that the DACHbox-N domain interacts with DNA [13], [28]. In DACHbox-N DHD, the surface composed of helix α2 and β4 and the loop between helix α3 and strand β5 is positively charged (Fig. 2B). This surface correlates with the DNA-interaction face of winged-helix motifs and was suggested to be the site of DNA binding for DACHbox-N. In both Ski-DHD and SnoN-DHD, the electrostatic potential of the corresponding surface is more neutral and a role in DNA-binding of these two proteins thus seems less likely.

### Conserved residues reveal a putative protein-interaction groove

**Figure 3:**
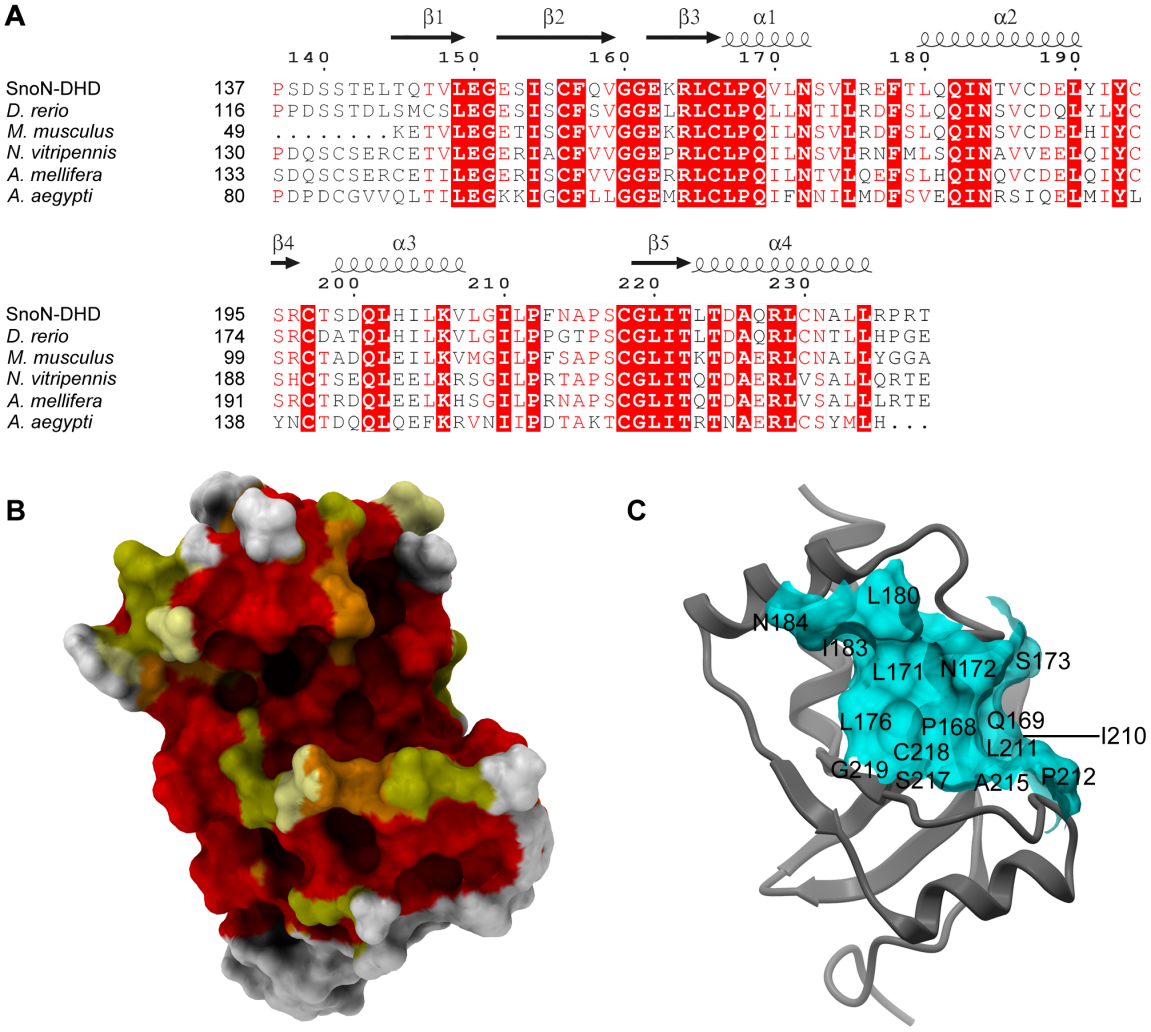
Conserved regions in SnoN-DHD. **Panel A**: Sequence alignment between human SnoN-DHD and five orthologous proteins. The sequence of SnoN-DHD corresponds to the protein fragment used in structural determination. The Genbank ID of the five orthologous sequences are: *Danio rerio* (gi:190338009), *Mus musculus* (gi:113205055), *Nasonia vitripennis* (gi: 156550231), *Apis mellifera* (gi: 110748778), and *Aedes aegypti* (gi: 157124676). Secondary structure elements and numbering of SnoN-DHD are depicted above the alignment. Alignment was generated by ClustalW [41] and colored by ESPript [40]. **Panel B**: Conserved residues on the surface of SnoN-DHD. The molecular surface of SnoN-DHD is colored according to the sequence conservation from the alignment in Fig 3A. Residues are colored in red and orange if they are strictly conserved in six or five sequences, respectively. Yellow and pale yellow represent residues that are similar in six and five sequences, respectively. Other residues are colored in white. SnoN-DHD is in the same orientation as in Fig 2A. **Panel C**: Conserved groove on the surface of SnoN-DHD. Structure of SnoN-DHD is represented in ribbon mode. The molecular surface of the conserved groove mentioned in the text is displayed and colored in light blue. It is composed of the following residues: Leu167, Pro168, Gln169, Leu171, Asn172, Ser173, Leu 180, Ile183, Asn184, Ile210, Leu211, Pro212, Ala215, Ser217, Cys218 and Gly219. The position of these residues on the surface is indicated by their corresponding one-letter label.

Based upon alignment of six orthologous proteins (Fig. 3A), a molecular surface representation of SnoN-DHD was rendered and colored according to the degree of sequence conservation (Fig. 3B). The figure reveals a highly conserved groove composed of helices α1 and α2, strand β5 and the loop connecting α3 to β5. Interestingly, this groove is located on the same face as the proposed DNA-binding region in DACHbox-N. It is composed of 16 residues and has a solvent-accessible surface area of about 690 Å^2^ according to ICM-Pro v.3.7-1e [29] (Fig. 3C). The size of this conserved groove corresponds to the buried surface area of an average protein-protein interface, that ranges between 600 to 1000 Å^2^ per subunit [30]. The groove is composed of aliphatic residues (Leu and Ile), non-charged polar residues, two prolines and an alanine. With the exception of the alanine and prolines, this composition is characteristic of a protein-protein interacting surface [31]. Depletion of Glu, Asp and Lys relative to the total composition of the solvent-exposed residues is a common feature of protein-protein interaction surfaces [31] and it can be noted that the conserved groove of SnoN-DHD lack such residues. Moreover, protein-protein interfaces appear to be enriched in Leu and Ile but not in Val and Ala. Leu and Ile are found in the SnoN-DHD groove but not Val and only one Ala. Taken together, the properties of this groove fit well with those of an average protein-protein interaction surface. Other conserved residues on the SnoN-DHD surface are mostly located on helix α4 and in the loop between β2 and β3 but do not form any patches big enough to mediate extended protein-protein or protein-DNA contacts.

### Open and tight states of the dachshund homology domain

**Figure 4:**
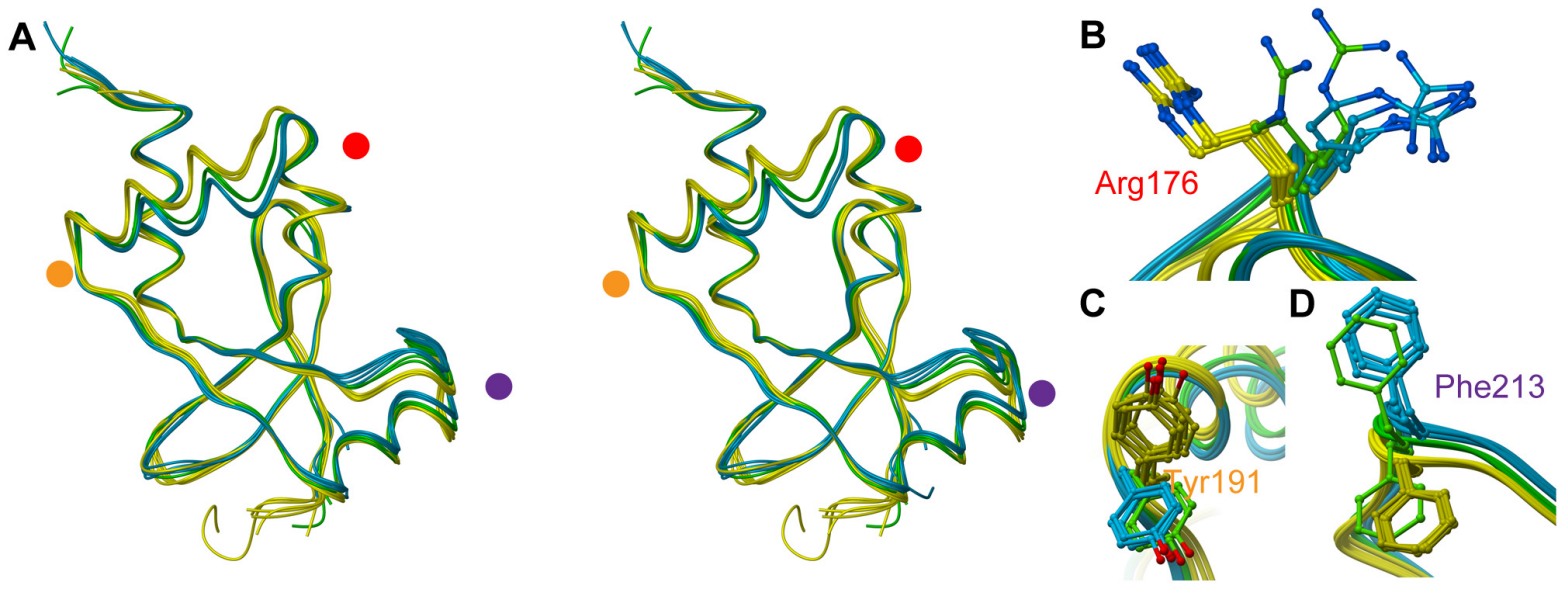
Conformational changes among the SnoN-DHD monomers. **Panel A**: Superposition of the twelve monomers of SnoN-DHD present in the asymmetric unit (stereo view). Chains A, B, C, D, E and G are colored in yellow, chains F, I, K and L in blue, and chains H and J in green. The red, orange and violet dots represent the location of Arg176, Tyr191 and Phe213, respectively. **Panels B–D**: Side-chain conformational changes of Arg176, Tyr191 and Phe213.

A superimposition of the twelve different monomers of SnoN-DHD present in the asymmetric unit reveals variability in the overall structure of the different monomers (Fig. 4A). The variations are localized primarily to secondary-structure elements bordering the conserved groove, *i.e.* helix α2 and the loop connecting helix α3 to strand β5. The most variable regions between the different monomers are in the N-terminus of helix α2 where the Cα of Asn184 has moved 2.8 Å, and in the α3-β5 connecting loop where the Cα of Pro216 has moved 2.8 Å. The shift of these regions causes the gap of the conserved groove to increase or decrease. Interestingly, the 12 different monomers in the SnoN asymmetric unit group into one of two basic conformations: an open conformation represented by chains A, B, C, D, E and G (colored yellow in Fig. 4) and a tight conformation represented by chains F, I, K and L (colored blue in Fig. 4). Chains H and J (green) are somewhat intermediate but closer to a tight conformation

The shift of the fold from open to tight conformation is accompanied by a number of side-chain reorientations in the structural elements flanking the conserved groove (Fig. 4B–D). For instance, the shift of the N-terminal part of the α2 helix towards the α3 helix closes the gap between the two, where α1 helix moves outwards. The conformational shifts of helices α1 and α2 are coupled to a motion of the connecting stretch wherein Arg176 displays a φ-angle rotation as well as side-chain flipping (Fig. 4B). On the C-terminal side of helix α2, where almost no backbone shift is seen, Tyr191 rotates 180 degrees around its χ1-angle (Fig. 4C). On the opposite side of the groove, in the loop connecting helix α3 and strand β5, only small backbone motions are seen but here Phe213 flips its aromatic ring 180 degrees towards the backbone of the loop (Fig. 4D). Within the crystal lattice, the Arg176, Tyr191 and Phe213 side-chains are involved in different intermolecular contacts in the two conformations. Although crystal packing is likely to stabilize the individual open and tight conformations, the crystal environment is not identical for all open-conformation chains, or for all tight-conformation chains. Together this suggests that there is a close equilibrium between the two conformations.

**Figure 5:**
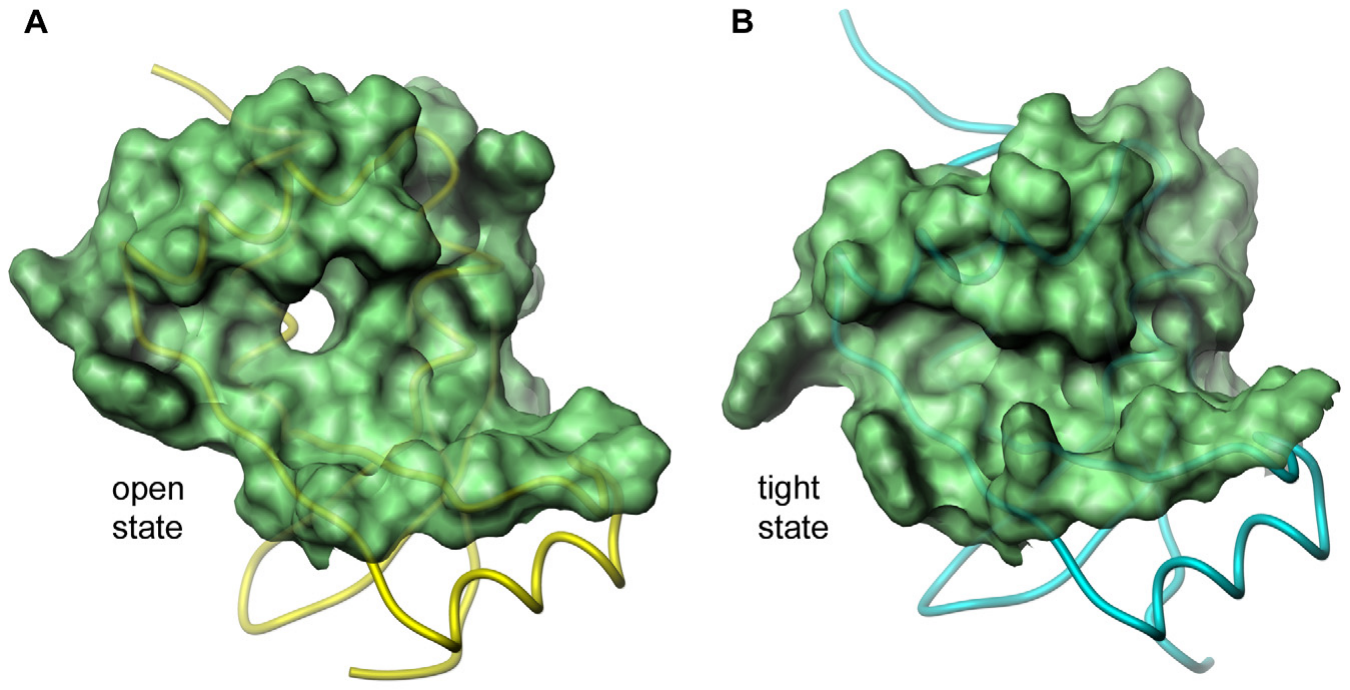
Open and tight conformation of SnoN-DHD: molecular surface representation. **Panel A**: SnoN chain C. Backbone of SnoN-DHD chain C is colored in yellow. Molecular surface of residues form the conserved groove and involved in conformational change is displayed in green. **Panel B**: SnoN chain F. Same as Panel A except that blue was used as a color for the backbone of chain F.

The gap of the open and tight conformations differs in width between approximately 13 and 7 Å, which is well suited to accommodate a protein helix or loop (Fig. 5). The open and tight conformations could thus well be part of an induced-fit mechanism allowing SnoN-DHD to increase interactions with a protein partner in the conserved groove.

**Figure 6:**
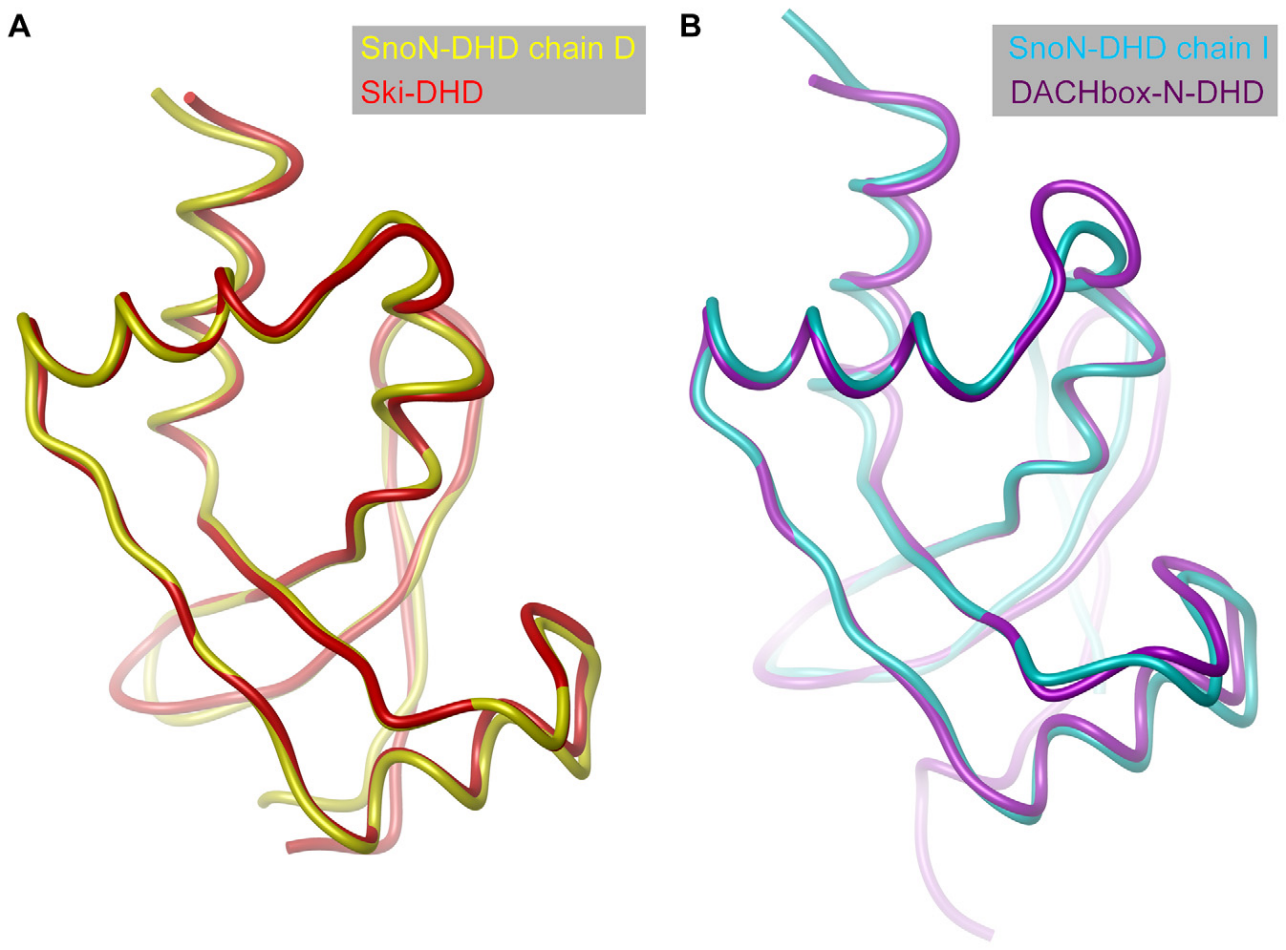
Superposition of SnoN-DHD, Ski-DHD and DACHbox-N. **Panel A**: Superposition of open conformation SnoN-DHD (chain D) and Ski-DHD. SnoN- and Ski-DHD are colored in yellow and red, respectively. Representation of Ski-DHD was limited to residues 97 to 192. **Panel B**: Superposition of tight conformation SnoN-DHD (chain I) and DACHbox-N. SnoN-DHD and DACHbox-N are colored respectively in blue and violet.

Interestingly, the major differences in backbone conformation of the previously determined DHD structures, *i.e.* from human Ski [12] and DACHbox-N [13], are located to the α2 helix and the α3-β5 connecting loop. These different conformations matches very well the open and tight conformations of SnoN-DHD (Fig. 6). The best superimposition between Ski-DHD and SnoN-DHD is achieved with chain D of SnoN-DHD (Fig. 6A, r.m.s.d. of 0.80 Å for 94 aligned Cα), whereas the best superimposition with the DACHbox-N is obtained with chain I of SnoN-DHD (Fig. 6B, r.m.s.d. of 1.17 Å for 92 aligned Cα). The Ski-DHD structure thus displays an open conformation while the DACHbox-N displays a tight conformation, as compared to SnoN-DHD. This finding suggests the existence of a general flexibility within the DHD fold, rather than an intrinsic structural difference between the Dachshund domains from these different proteins. Although both Ski- and SnoN-DHD are protein-interacting domains, their DHD fold has retained the interaction site from their DNA-interacting winged-helix relatives.

The SnoN-DHD structure presented here and the comparison with related structures strongly suggest the existence of a conserved and flexible groove which constitutes a likely protein-interaction surface. The flexibility of this groove might be necessary to accommodate different binding partners, such as N-CoR or Ski-interacting protein (Skip) [32]. Future structural and biochemical studies of the DHD family members with their potential interaction partners should allow the evaluation of this model.

## Materials and Methods

### Protein expression and purification

Human *sno* (*skil*) cDNA was obtained from Mammalian Gene Collection (accession no. BC059386). The sequence encoding SnoN residues 137–238 was amplified by PCR and inserted into pNIC28-Bsa4 by ligation independent cloning. The expression construct included a TEV protease-cleavable N-terminal hexahistidine tag. Protein expression in *Escherichia coli* strain BL21(DE3) R3 pRARE was done in a LEX system (Harbinger Biotechnology and Engineering) using Terrific Broth medium supplemented with 8 g/l glycerol, 34 µg/ml chloramphenicol and 50 µg/ml kanamycin, induction with 0.5 mM IPTG, and over night culture at 18°C. Cells were harvested by centrifugation 4,400 g for 10 min at 4°C and resuspended in 50 mM HEPES pH 7.8, 500 mM NaCl, 10 mM imidazole, 10% glycerol, 0.5 mM TCEP, and Complete EDTA-free Protease Inhibitor (Roche Biosciences). Cells were lysed by a freeze/thaw cycle followed by addition of benzonase (Novagen) and sonication (Sonics VibraCell). Lysates were centrifuged at 49,000 g for 20 min at 4°C. The supernatants were decanted and filtered.

Filtered lysates of cells expressing SnoN^137–238^ were loaded onto HiTrap Chelating HP columns (GE Healthcare) in buffer 1 (30 mM HEPES pH 7.5, 500 mM NaCl, 10 mM imidazole, 10% glycerol, and 0.5 mM TCEP). The columns were washed with buffer 1 containing 25 mM imidazole. Bound protein was eluted with buffer 1 containing 500 mM imidazole, and loaded onto a HiLoad 16/60 Superdex-75 column (GE Healthcare) pre-equilibrated with buffer 2 (20 mM HEPES pH 7.5, 300 mM NaCl, 10% glycerol, 2 mM TCEP). Fractions containing target protein were pooled, and the N-terminal hexahistidine tag was removed by incubation with His-tagged TEV protease (molar ratio 30∶1) over night at 4°C, followed by passage over a 1 ml HiTrap Chelating HP column pre-equilibrated with buffer 1 with 25 mM imidazole. The cleaved protein was concentrated and the buffer was changed to buffer 2 using a centrifugal filter device with 5,000 NMWL. The final protein concentration was determined to 19.6 mg/ml in a volume of 0.7 ml. Aliquots were flash-frozen and stored at −80°C. The identity of the protein was verified by time-of-flight mass spectrometry analysis.

### Crystallization, data collection, structure solution and refinement

Crystals of SnoN-DHD were obtained by the sitting drop vapour diffusion method in a 96-well plate. 0.1 µl of the protein solution (19.6 mg/ml) was mixed with 0.2 µl of well solution consisting of 0.2 M ammonium nitrate and 20% (w/v) PEG 3350. The plate was incubated at 4°C and rhombohedral crystals appeared after 15 to 29 days. After 73 days, the crystal was transferred to cryo solution consisting of 20 mM HEPES, 300 mM NaCl, 20% glycerol, 0.2 M ammonium nitrate and 20% (w/v) PEG 3350, pH 7.5, and flash-frozen in liquid nitrogen.

Diffraction data to 2.45 Å resolution were collected at ESRF Grenoble, France (beamline ID 29). Data was integrated and scaled using XDS and XSCALE [33]. PHASER [34] found 7 correct monomers in the asymmetric unit using the structure of the Dachshund-homology domain of human SKI (PDB-code: 1SBX) [12] as a probe. This solution was refined in CNS [35] using simulated annealing. The resulting monomer model was used as a probe in a new query in PHASER and resulted in the discovery of two more monomers. Analysis of the crystallographic contacts allowed to build 3 additional monomers in the density map with Coot [36], resulting in the final asymmetric unit of 12 monomers. Data in the interval 19.95–2.45 Å resolution was used for refinement. Iterative cycles of manual building in both Coot and O [37] and restrained refinement in REFMAC5 [38] were performed. TLS refinement was included in the last steps of the refinement process. According to the results provided by the TLSMD server [39], 11 monomers were divided into 3 TLS groups, 4 TLS groups being used for monomer E. For further details, see Table S1. The atomic coordinates and structure factors were deposited to the Protein Data Bank, www.rcsb.org (PDB entry code 3EQ5). Sequence alignments were obtained using ESPript [40]. Structural images and iSee datapack were rendered using ICM-Pro v.3.7-1e [29].

## Supporting Information

Datapack S1 Standalone iSee datapack - contains the enhanced version of this article for use offline. This file can be opened using free software available for download at http://www.molsoft.com/icm_browser.html.
(ICB)

Table S1 Data collection and refinement statistics for SnoN-DHD.
(0.12 MB DOC)

Text S1 Instructions for installation and use of the required web plugin (to access the online enhanced version of this article).
(0.44 MB PDF)

## References

[1] Luo K, Stroschein SL, Wang W, Chen D, Martens E (1999). The Ski oncoprotein interacts with the Smad proteins to repress TGFbeta signaling.. Genes Dev.

[2] Stroschein SL, Wang W, Zhou S, Zhou Q, Luo K (1999). Negative feedback regulation of TGF-beta signaling by the SnoN oncoprotein.. Science.

[3] Dahl R, Kieslinger M, Beug H, Hayman MJ (1998). Transformation of hematopoietic cells by the Ski oncoprotein involves repression of retinoic acid receptor signaling.. Proc Natl Acad Sci U S A.

[4] Ritter M, Kattmann D, Teichler S, Hartmann O, Samuelsson MK (2006). Inhibition of retinoic acid receptor signaling by Ski in acute myeloid leukemia.. Leukemia.

[5] Tokitou F, Nomura T, Khan MM, Kaul SC, Wadhwa R (1999). Viral ski inhibits retinoblastoma protein (Rb)-mediated transcriptional repression in a dominant negative fashion.. J Biol Chem.

[6] Zheng G, Teumer J, Colmenares C, Richmond C, Stavnezer E (1997). Identification of a core functional and structural domain of the v-Ski oncoprotein responsible for both transformation and myogenesis.. Oncogene.

[7] Nomura T, Khan MM, Kaul SC, Dong HD, Wadhwa R (1999). Ski is a component of the histone deacetylase complex required for transcriptional repression by Mad and thyroid hormone receptor.. Genes Dev.

[8] Stavnezer E, Gerhard DS, Binari RC, Balazs I (1981). Generation of transforming viruses in cultures of chicken fibroblasts infected with an avian leukosis virus.. J Virol.

[9] Li Y, Turck CM, Teumer JK, Stavnezer E (1986). Unique sequence, ski, in Sloan-Kettering avian retroviruses with properties of a new cell-derived oncogene.. J Virol.

[10] Nomura N, Sasamoto S, Ishii S, Date T, Matsui M (1989). Isolation of human cDNA clones of ski and the ski-related gene, sno.. Nucleic Acids Res.

[11] Pearson-White S (1993). SnoI, a novel alternatively spliced isoform of the ski protooncogene homolog, sno.. Nucleic Acids Res.

[12] Wilson JJ, Malakhova M, Zhang R, Joachimiak A, Hegde RS (2004). Crystal structure of the dachshund homology domain of human SKI.. Structure.

[13] Kim SS, Zhang RG, Braunstein SE, Joachimiak A, Cvekl A (2002). Structure of the retinal determination protein Dachshund reveals a DNA binding motif.. Structure.

[14] Heyman HC, Stavnezer E (1994). A carboxyl-terminal region of the ski oncoprotein mediates homodimerization as well as heterodimerization with the related protein SnoN.. J Biol Chem.

[15] Bottomley MJ, Collard MW, Huggenvik JI, Liu Z, Gibson TJ (2001). The SAND domain structure defines a novel DNA-binding fold in transcriptional regulation.. Nat Struct Biol.

[16] Wu JW, Krawitz AR, Chai J, Li W, Zhang F (2002). Structural mechanism of Smad4 recognition by the nuclear oncoprotein Ski: insights on Ski-mediated repression of TGF-beta signaling.. Cell.

[17] Deheuninck J, Luo K (2009). Ski and SnoN, potent negative regulators of TGF-beta signaling.. Cell Res.

[18] Cohen SB, Zheng G, Heyman HC, Stavnezer E (1999). Heterodimers of the SnoN and Ski oncoproteins form preferentially over homodimers and are more potent transforming agents.. Nucleic Acids Res.

[19] Arndt S, Poser I, Schubert T, Moser M, Bosserhoff AK (2005). Cloning and functional characterization of a new Ski homolog, Fussel-18, specifically expressed in neuronal tissues.. Lab Invest.

[20] Arndt S, Poser I, Moser M, Bosserhoff AK (2007). Fussel-15, a novel Ski/Sno homolog protein, antagonizes BMP signaling.. Mol Cell Neurosci.

[21] Colmenares C, Stavnezer E (1989). The ski oncogene induces muscle differentiation in quail embryo cells.. Cell.

[22] Boyer PL, Colmenares C, Stavnezer E, Hughes SH (1993). Sequence and biological activity of chicken snoN cDNA clones.. Oncogene.

[23] Fumagalli S, Doneda L, Nomura N, Larizza L (1993). Expression of the c-ski proto-oncogene in human melanoma cell lines.. Melanoma Res.

[24] Massague J (1998). TGF-beta signal transduction.. Annu Rev Biochem.

[25] Heldin CH, Miyazono K, ten Dijke P (1997). TGF-beta signalling from cell membrane to nucleus through SMAD proteins.. Nature.

[26] Krakowski AR, Laboureau J, Mauviel A, Bissell MJ, Luo K (2005). Cytoplasmic SnoN in normal tissues and nonmalignant cells antagonizes TGF-beta signaling by sequestration of the Smad proteins.. Proc Natl Acad Sci U S A.

[27] Greene LH, Lewis TE, Addou S, Cuff A, Dallman T (2007). The CATH domain structure database: new protocols and classification levels give a more comprehensive resource for exploring evolution.. Nucleic Acids Res.

[28] Ikeda K, Watanabe Y, Ohto H, Kawakami K (2002). Molecular interaction and synergistic activation of a promoter by Six, Eya, and Dach proteins mediated through CREB binding protein.. Mol Cell Biol.

[29] Abagyan RAea (1994). ICM: a new method for protein modeling and design: applications to docking and structure prediction from the distorted native conformation.. J Comp Chem.

[30] Hwang H, Pierce B, Mintseris J, Janin J, Weng Z (2008). Protein-protein docking benchmark version 3.0.. Proteins.

[31] Janin J, Bahadur RP, Chakrabarti P (2008). Protein-protein interaction and quaternary structure.. Q Rev Biophys.

[32] Dahl R, Wani B, Hayman MJ (1998). The Ski oncoprotein interacts with Skip, the human homolog of Drosophila Bx42.. Oncogene.

[33] Kabsch W (1993). Automatic processing of rotation diffraction data from crystals of initially unknown symmetry and cell constants.. J Appl Cryst.

[34] McCoy AJ, Grosse-Kunstleve RW, Adams PD, Winn MD, Storoni LC (2007). Phaser crystallographic software.. J Appl Crystallogr.

[35] Brunger AT (2007). Version 1.2 of the Crystallography and NMR system.. Nat Protoc.

[36] Emsley P, Lohkamp B, Scott WG, Cowtan K (2010). Features and development of Coot.. Acta Crystallogr D Biol Crystallogr.

[37] Jones DT (1999). Protein secondary structure prediction based on position-specific scoring matrices.. J Mol Biol.

[38] Murshudov GN, Vagin A, Dodson EJ (1997). Refinement of macromolecular structures by the maximum-likelihood method.. Acta Crystallogr D Biol Crystallogr.

[39] Painter J, Merritt EA (2006). TLSMD web server for the generation of multi-group TLS models.. Journal of Applied Crystallography.

[40] Gouet P, Courcelle E, Stuart DI, Metoz F (1999). ESPript: analysis of multiple sequence alignments in PostScript.. Bioinformatics.

[41] Chenna R, Sugawara H, Koike T, Lopez R, Gibson TJ (2003). Multiple sequence alignment with the Clustal series of programs.. Nucleic Acids Res.

