# Supplementary material for: The Crystal Structure of the Dachshund Domain of Human SnoN Reveals Flexibility in the Putative Protein Interaction Surface
Source: PLoS One. 2010 Sep 23;5(9):e12907. doi: 10.1371/journal.pone.0012907 (PMC2944819; doi:10.1371/journal.pone.0012907)
Supplement: Table S1 — Data collection and refinement statistics for SnoN-DHD. (0.12 MB DOC) [file pone.0012907.s002.doc]

**Table S1.** Data collection and refinement statistics for SnoN-DHD

| **PDB Code** | **3EQ5** |
| --- | --- |
| **Data collection** |  |
| **X-ray source** | **ESRF-ID29** |
| Wavelength (Å) | 0.97623 |
| Spacegroup | C2 |
| Cell dimensions |  |
| *a, b, c* (Å) | 210.82, 70.12,116.28 |
| *α, β, γ* (º) | 90.00, 100.95, 90.00 |
| Resolution (Å)* | 19.83-2.45 (2.51-2.45) |
| R_meas_ (%)† | 7.4 (59.7) |
| I/σ(I) | 12.0 (2.3) |
| Completeness (%) | 100.0 (100.0) |
| Redundancy | 2.6 (2.6) |
| **Refinement** |  |
| Resolution (Å) | 19.83-2.45 |
| No. reflections (test set) | 57326 (3018) |
| R_work_/R_free_ (%) | 23.2/27.4 |
| No. atoms |  |
| Protein | 9094 |
| Water | 343 |
| R.m.s. deviations |  |
| Bond lengths (Å) | 0.006 |
| Bond angles (º) | 0.808 |
| Ramachandran plot (% residues)± |  |
| Favored | 97.2 |
| Allowed | 2.8 |

* Highest resolution shell shown between parentheses.

† R_meas_ is defined according to Diederichs and Karplus (Diederichs K, Karplus PA. (1997) Improved R-factors for diffraction data analysis in macromolecular crystallography. Nat Struct Biol 4: 269-275).

± Calculated with Molprobity (Davis IW *et al*. (2007) MolProbity: all-atom contacts and structure validation for proteins and nucleic acids. Nucleic Acids Res 35(Web Server issue): W375-383).
